# Cytokines IL-17, TNF and IFN-γ Alter the Expression of Antimicrobial Peptides and Proteins Disparately: A Targeted Proteomics Analysis using SOMAscan Technology

**DOI:** 10.3390/vaccines6030051

**Published:** 2018-08-07

**Authors:** Anthony Altieri, Hadeesha Piyadasa, Breann Recksiedler, Victor Spicer, Neeloffer Mookherjee

**Affiliations:** 1 Department of Immunology, University of Manitoba, Winnipeg, MB R3E0T5, Canada; umaltier@myumanitoba.ca (A.A.); Hadeesha.piyadasa@umanitoba.ca (H.P.); recksie4@myumanitoba.ca (B.R.); 2 Manitoba Center for Proteomics and Systems Biology, Department of Internal Medicine, University of Manitoba, Winnipeg, MB R3E3P4, Canada; Victor.Spicer@umanitoba.ca

**Keywords:** Host defence peptides, antimicrobial peptides, inflammation, cytokines, bronchial epithelial cells

## Abstract

Antimicrobial peptides, also known as host defence peptides, are immunomodulatory molecules required to resolve infections. Antimicrobial peptides and proteins (APPs) are important in the control of infections in the lungs. Despite evidence that APPs exhibit a wide range of immune functions and modulate inflammation, the effect of inflammatory cytokines on the expression of APPs is not completely defined. In this study, we profiled the expression of 39 different APPs in human bronchial epithelial cells (HBEC) using Slow Off-rate Modified Aptamer (SOMAmer)-based protein array, in the presence and absence of three different inflammatory cytokines (IL-17, TNF and IFN-γ). Expression of 13 different APPs was altered in response to IL-17, TNF or IFN-γ. Independent validations of selected proteins from the proteomics screen i.e., those that were significantly enhanced by >2-fold change (*p* < 0.01) using western blots conclusively demonstrated that inflammatory cytokines alter the expression of APPs differentially. For example, the abundance of cathepsin S was enhanced by only IFN-γ, whereas lipocalin-2 was increased by IL-17 alone. Abundance of elafin increased in presence of IL-17 or TNF, but decreased in response to IFN-γ. Whereas the abundance of cathepsin V decreased following stimulation with IL-17, TNF and IFN-γ. The results of this study demonstrate that inflammatory cytokines alter the expression of APPs disparately. This suggests that the composition of the inflammatory cytokine milieu may influence APPs abundance and thus alter the processes required for infection control and regulation of inflammation in the lungs.

## 1. Introduction

Antimicrobial proteins and peptides (APPs) are cationic molecules that are effective in resolving infections either by direct effects on the pathogen or indirectly by modulating host immune functions [1,2,3]. Antimicrobial peptides, also known as host defence peptides, are amphipathic peptides, typically containing less than 50 amino acids with a net positive charge from +2 to +9. These peptides can be broadly classified into four structural classes; (1) β-sheet structures with disulfide bonds, (2) extended structures, (3) loop structures with one disulfide bond and (4) amphipathic α-helices. Antimicrobial proteins are larger proteins that contain multiple polypeptide subunits and exhibit catalytic activity [4]. APPs are found in a wide variety of complex life forms, including insects, plants and animals [3,5,6]. APPs are produced primarily by immune cells such as monocytes/macrophages and neutrophils [1], as well as by epithelial cells at mucosal surfaces [7]. APPs exhibit a wide range of immunomodulatory functions; alter immune signaling events induced by pathogens and inflammatory cytokines, contribute to maintaining immune homeostasis, and regulate the inflammatory process [4,7,8,9]. Despite evidence that the expression of APPs can be altered in an inflammatory event [10], and that APPs can in turn modulate the process of inflammation [4,7,8,9], the effect of inflammatory cytokines on APP expression has not been completely defined.

APPs play a critical role in the control of infections in the lungs [2,4]. These molecules exhibit immunomodulatory functions in the lungs, such as facilitating phagocytosis and altering innate immune signaling events induced in response to pathogens and inflammation [2]. It has been shown that expression of specific APPs, particularly certain host defence peptides, increases during pneumonia [10]. Previous studies have demonstrated that the expression of specific APPs in the lungs change in response to inflammatory stimuli such as infection, allergens, air pollution and in chronic inflammatory disease [11,12,13,14,15,16]. APPs are expressed in the epithelial lining of the lungs [2,4], where they are produced by bronchial epithelial cells [2,17]. Therefore, human bronchial epithelial cells (HBECs) are ideal to examine the effect of inflammatory cytokines on APPs expression levels. In this study, we examined the expression of APPs in HBEC using a targeted proteomics approach, following stimulation with inflammatory cytokines IL-17, TNF and IFN-γ, all known to mediate airway inflammation.

IL-17, TNF and IFN-γ are critical pro-inflammatory cytokines that are elevated in airway inflammation [18,19,20,21,22]. IL-17 contributes to pulmonary host defense, and subsequently enhances inflammation through the production of pro-inflammatory signals, which promote neutrophil mobilization and the expression of antimicrobial factors in the lungs [23,24]. TNF functions in a similar manner, promoting neutrophil mobilization and recruitment, and enhancing inflammation in the lungs [25]. Whereas, IFN-γ is capable of activating monocytes and macrophages, mast cells, dendritic cells, eosinophils, and basophils [26]. Despite the advancements in understanding how IL-17, TNF and IFN-γ contribute to airway inflammation through immune cell activation and recruitment, the effect of these cytokines on APPs production in the lungs is not completely defined. Therefore, in this study we used a Slow Off-rate Modified Aptamer (SOMAmer^®^)-based protein array (SOMAscan^®^ platform) to profile the expression of 39 APPs in HBEC, in response to IL-17, TNF and IFN-γ. SOMAscan^®^ is an aptamer based, highly sensitive, proteomics platform that can monitor the protein expression of more than 1300 proteins in an array [27]. We demonstrated that the abundance of 13 different APPs significantly altered in response to stimulation with either IL-17, TNF, or IFN-γ in HBEC. Overall, the APP expression profiles were similar in cells in response to TNF or IL-17, whereas IFN-γ mediated a distinct expression profile compared to the other two cytokines. The results in this study highlight the disparate alteration of APP expression by inflammatory cytokines IL-17, TNF and IFN-γ, in bronchial epithelial cells. These results suggest that changes in specific APP abundance based on the composition of airway inflammatory milieu may affect the ability to resolve pulmonary infections.

## 2. Materials and Methods

### 2.1. Cell Culture

HBEC-3KT (ATCC^®^ CRL-4051™) were cultured in the airway epithelial cells basal medium (ATCC^®^ PCS-300-030™) and supplemented with bronchial epithelial cells growth kit (ATCC^®^ PCS-300-040™), according to instructions from ATCC^®^. HBECs were maintained at ~80% confluency to ensure epithelial morphology. HBECs were trypsinized with 1:3 dilution of 0.5% trypsin-EDTA (Invitrogen™, Life Technologies Inc, Burlington, ON, Canada) in PBS. The culture medium was changed to airway epithelial cells basal medium containing only 6 mM L-glutamine from the bronchial epithelial cells growth kit (and no other growth factors), to simulate a serum starvation condition 24 h prior to addition of the various stimulants. 

### 2.2. Reagents and Antibodies

Recombinant human cytokines TNF, IFN-γ and IL-17A/F were obtained from R&D Systems. Anti-human lipocalin-2, elafin, cathepsin S and cathepsin V antibodies were obtained from Abcam (Toronto, ON, Canada). Anti-human actin antibody was obtained from Millipore (Burlington, MA, USA). HRP-linked purified anti-rabbit IgG and anti-mouse IgG-secondary antibodies were obtained from Cell Signaling Technology, distributed by New England Biolabs (Pickering, ON, Canada). 

### 2.3. Slow Off-Rate Modified Aptamer (SOMAmer^®^)-based Protein Array

HBECs were stimulated with IL-17A/F (50 ng/mL), TNF (20 ng/mL), or IFN-γ (30 ng/mL), for 24 h. Total cell lysates were prepared in lysis buffer containing M-PER™ (Thermo Fisher Scientific, Burlington, ON, Canada) and HALT protease and phosphatase inhibitor cocktail (Thermo Fisher Scientific). Protein concentration was determined by micro BCA protein assay kit (Thermo Fisher Scientific) and 14 μg total protein per sample was used for the SOMAmer^®^-based protein arrays (SOMALogic, Inc., Boulder, CO, USA). The protein arrays used in the SOMAscan^®^ platform are made up of single strand DNA-based protein affinity reagents known as SOMAmer^®^ (Slow Off-rate Modified Aptamer) which are highly sensitive and specific for their protein targets. This approach uses the chemically modified nucleotides (SOMAmer^®^) to provide specific protein abundance using relative fluorescence units (RFU) on the arrays [27,28]. The SOMAmer^®^ protein arrays used were for >1300 protein targets. The arrays were processed and analyzed according to the manufacturer’s recommended protocol (SOMALogic, Inc., Boulder, CO, USA). In this study, we focused on the expression profile of APPs in the protein array (39 targets in the array were APPs). The RFU readout values were log2 transformed for differential analysis. 

### 2.4. Western Blots

Cell lysates were prepared in lysis buffer (PBS containing 1% (*v*/*v*) protease inhibitor cocktail (Cell Signaling Technology, Danvers, MA, USA) and 0.5% (*v*/*v*) nonidet-P40 (Sigma, Tokyo, Japan). Cell lysates (total protein 10 μg per sample) were resolved on 4–12% NuPAGE Bis-Tris gels (Invitrogen, Waltham, MA, USA) followed by transfer to nitrocellulose membranes (Millipore, Burlington, MA, USA). The membranes were subsequently blocked with TBST (20 mm Tris-HCl pH 7·5, 150 mm NaCl, 0·1% Tween-20) containing 5% (*w*/*v*) skimmed milk powder, and probed with various antibodies as indicated in TBST containing 2.5% (*w*/*v*) skimmed milk powder. Affinity purified HRP-linked secondary antibodies (Cell Signaling Technology) were used for detection, and the membranes were developed using the Amersham ECL detection system (GE Healthcare, Mississauga, ON, Canada) according to the manufacturer’s instructions.

### 2.5. ELISA

Tissue culture supernatants were centrifuged at 250× *g* for 5 min to obtain cell free samples, and the aliquots were stored at −20 °C for further use. Cytokine and chemokine concentrations were examined in the tissue culture supernatants using ELISA. Production of chemokines CXCL1 and CXCL8 were examined using antibody pairs from R&D Systems DuoSet kits as per the manufacturer’s instructions. Production of MCP-1 was monitored using an eBioscience ELISA kit as per the manufacturer’s instructions.

### 2.6. Statistical Analyses

One-way analysis of variance (ANOVA) was used to compare the expression values between the different conditions in the SOMAmer^®^-based protein arrays. Heat map was generated using Multi-Experiment Viewer Version 10.2. GraphPad PRISM 6 was used for statistical analysis by Mann-Whitney U test (*p* < 0.01) to compare independent protein expression in response to various stimulants relative to unstimulated cells, in western blot densitometry, and for ELISA results. A value of *p* < 0.01 was considered to be statistically significant for all experiments. 

## 3. Results

### 3.1. Chemokine Production in Response to IL-17, TNF and IFN-γ

**Figure 1:**
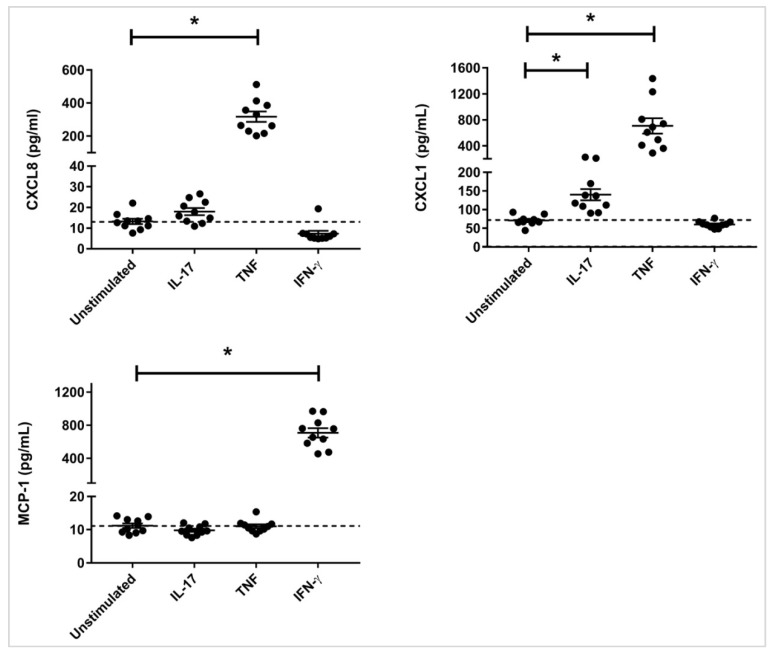
Chemokine production induced by IL-17, TNF and IFN-γ, HBECs were stimulated with either IL-17 (50 ng/mL), TNF (20 ng/mL), or IFN-γ (30 ng/mL). Tissue culture supernatants were monitored for the production of chemokines CXCL1, CXCL8, and MCP-1 by ELISA after 24 h. Data shown represents the mean ± standard error for 10 independent experiments. Mann-Whitney U test was used to determine statistical significance (* *p* < 0.01).

Pro-inflammatory mediators such as pathogen-derived ligands, allergens and inhaled pollutants can induce inflammatory cytokines IL-17, TNF and IFN-γ, as well as alter specific APP expression in the airways [11,12,13,14,15,16,29]. Therefore, we evaluated chemokine production as a read out for downstream response to cytokines IL-17, TNF and IFN-γ in HBEC. The cells were stimulated with IL-17A/F (50 ng/mL), TNF (20 ng/mL), or IFN-γ (30 ng/mL), for 24 h. The concentrations of the cytokines were selected as optimal based on previous studies [30,31]. IL-17 and TNF are known to stimulate neutrophil chemokines, and in contrast IFN-γ stimulates chemokines that attract monocytic cells [30,31,32,33]. Therefore, we evaluated the production of neutrophilic chemokines CXCL8 (IL-8) and CXCL1 (Gro-α), and monocyte chemoattractant protein-1 (MCP-1) in the tissue culture supernatants by ELISA. IL-17 significantly induced the production of CXCL1, and TNF induced the production of both CXCL8 and CXCL1, in HBEC after 24 h (Figure 1). In contrast, IFN-γ significantly induced the production of MCP-1 in HBEC after 24 h (Figure 1). Based on these results we selected the time point of 24 h for the proteomic screen in HBEC.

### 3.2. Differential APP Expression Profiles in Response to IL-17, TNF and IFN-γ

**Figure 2:**
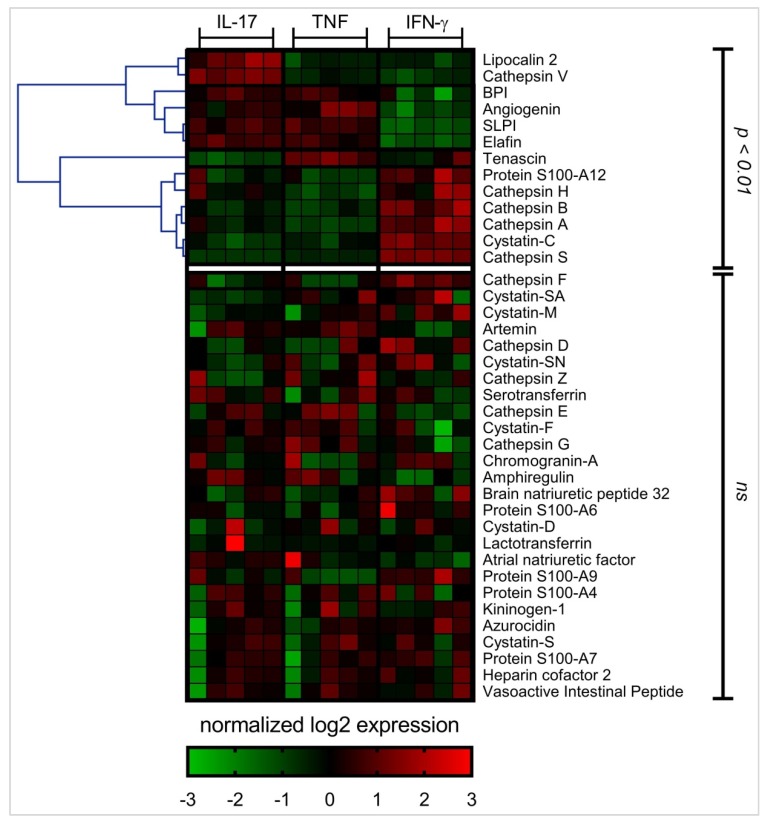
APPs expression profile mediated by IL-17, TNF and IFN-γ, HBECs were stimulated with either IL-17 (50 ng/mL), TNF (20 ng/mL), or IFN-γ (30 ng/mL) for 24 h. Equivalent amount of protein from each total cell lysate was processed using SOMAmer^®^-based protein arrays. The RFU readout values were log2 transformed for differential analysis. Log2 expression values were normalized per row in the heat map to yield a consistent dynamic range for visualization. One-way analysis of variance (ANOVA) was used to compare RFU values between the different conditions in the SOMAmer^®^-based protein arrays, and *p* < 0.01 was considered to be statistically significant. Heat map was generated using Multi-Experiment Viewer Version 10.2.

**Figure 3:**
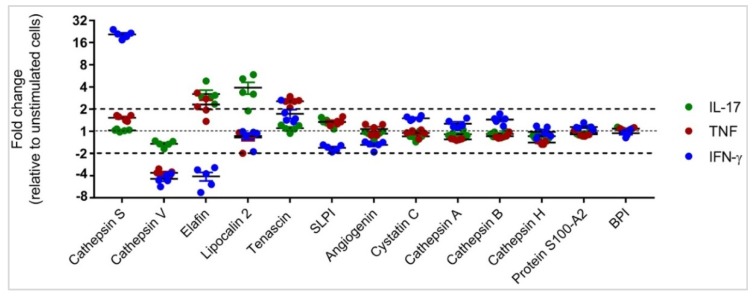
Relative abundance of APPs significantly altered by cytokines IL-17, TNF and/or IFN-γ, HBECs were stimulated with either IL-17 (50 ng/mL), TNF (20 ng/mL), or IFN-γ (30 ng/mL) for 24 h. Equivalent amount of protein from each total cell lysate was processed using SOMAmer^®^-based protein arrays. The RFU readout values were log2 transformed for differential analysis. Pair-wise differential analysis was performed to select proteins that were statistically significant using one-way analysis of variance (ANOVA) and *p* < 0.01 was considered to be statistically significant. APPs with expression values ≥ 2-fold relative to unstimulated cells (*p* < 0.01) were selected for further validation. Each dot represents the expression value from an independent cell lysate, the plots show mean ± standard error.

The abundance of 39 different APPs was examined in HBEC lysates following stimulation with either IL-17A/F (50 ng/mL), TNF (20 ng/mL), or IFN-γ (30 ng/mL) using the SOMAscan^®^ platform, after 24 h. Differential analysis was performed on log2 protein expression values using ANOVA (*p* < 0.01) to identify APPs that were differentially expressed in response to IL-17, TNF, or IFN-γ, compared to unstimulated cells (Figure 2). HBECs stimulated with IL-17 and TNF showed similar APP expression profiles, whereas IFN-γ mediated a distinct expression profile of APPs (Figure 2). The abundance of 13 different APPs was significantly (*p* < 0.01) altered in response to cytokine stimulation compared to unstimulated cells (Figure 2). We further sorted these 13 APPs based on expression values compared to unstimulated cells. This demonstrated that the abundance of five specific APPs (cathepsin S, cathepsin V, elafin, lipocalin-2 and tenascin) was altered by more than 2-fold (*p* < 0.01) in response to IL-17, TNF or IFN-γ (Figure 3), compared to unstimulated cells. Cathepsin S abundance was increased by ~20-fold in response to IFN-γ alone, compared to unstimulated cells (Figure 3). In contrast, cathepsin V protein abundance was decreased in response to all three cytokines, with TNF and IFN-γ resulting in decrease in protein abundance by > 4-fold, whereas that by IL-17 was less than 2-fold, compared to unstimulated cells (Figure 3). Elafin protein abundance was increased ≥2-fold in response to IL-17 and TNF, and in contrast IFN-γ decreased elafin abundance by ~4-fold, compared to unstimulated cells (Figure 3). Lipocalin-2 protein abundance was increased ~4-fold in response to IL-17 uniquely, whereas tenascin protein abundance was increased ~3-fold by TNF uniquely. These results indicated that inflammatory cytokines such as IL-17, TNF and IFN-γ mediate disparate alteration of APP protein expression profiles in HBEC. The IL-17- and TNF-mediated APP profiles are similar, while the IFN-γ-mediated APP expression profile is distinct.

### 3.3. Independent Validation of Specific APP Production in HBEC

**Figure 4:**
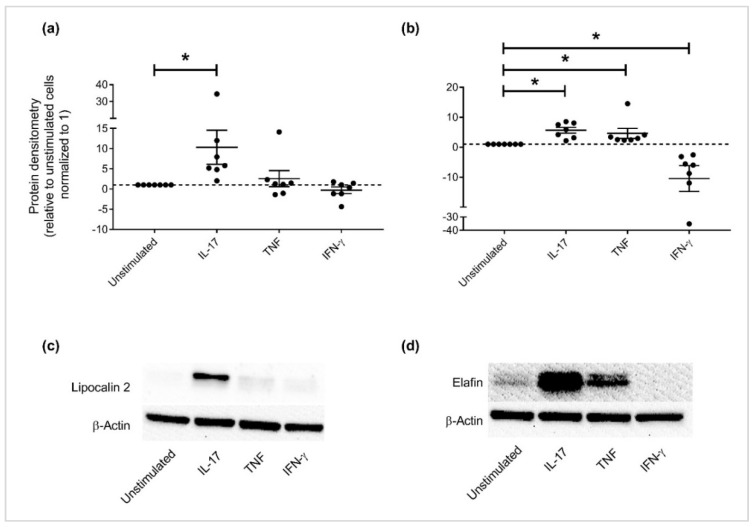
Expression of lipocalin-2 and elafin altered by cytokines IL-17, TNF and/or IFN-γ, HBECs were stimulated with either IL-17 (50 ng/mL), TNF (20 ng/mL), or IFN-γ (30 ng/mL) for 24 h. Total cell lysates (10 μg total protein per sample) were probed in immunoblots to assess the abundance of (**a**) lipocalin-2 and (**b**) elafin, and quantified by densitometry. Abundance of β-actin was used for normalization of protein load across samples. Y-axis represents relative band intensity compared to unstimulated cells normalized to 1. Each dot represents an independent experiment, with the mean (from seven independent experiments) ± standard error. Mann-Whitney U test was used for statistical analysis (* *p* < 0.01). Representative immunoblot shown for (**c**) lipocalin-2 and (**d**) elafin.

**Figure 5:**
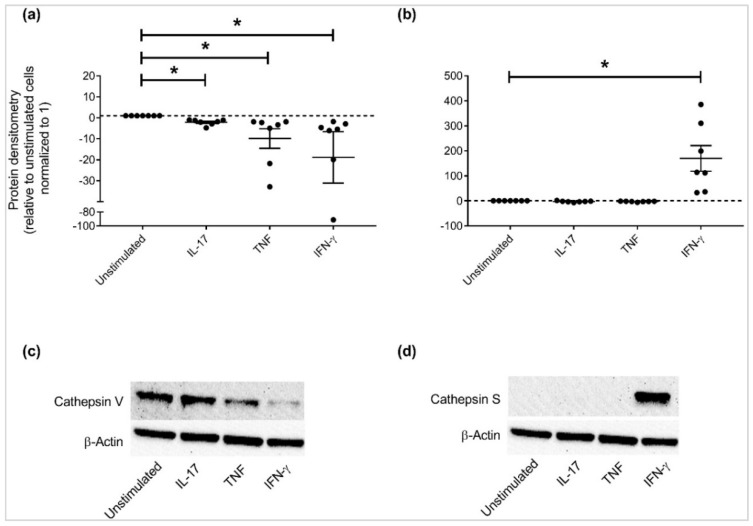
Expression of cathepsins altered by cytokines IL-17, TNF and/or IFN-γ, HBECs were stimulated with either IL-17 (50 ng/mL), TNF (20 ng/mL), or IFN-γ (30 ng/mL) for 24 h. Total cell lysates (10 μg total protein per sample) were probed in immunoblots to assess the abundance of (**a**) Cathepsin V and (**b**) Cathepsin S, and quantified by densitometry. Abundance of β-actin was used for normalization of protein load across samples. Y-axis represents relative band intensity compared to unstimulated cells normalized to 1. Each dot represents an independent experiment (from seven independent experiments), graphs show mean ± standard error. Mann-Whitney U test was used for statistical analysis (* *p* < 0.01). Representative immunoblot shown for (**c**) Cathepsin V and (**d**) Cathepsin S.

APPs that were altered ≥ 2-fold relative to unstimulated cells (*p* < 0.01) i.e., cathepsin S, cathepsin V, elafin, lipocalin-2 and tenascin (Figure 3) were selected for further independent validation. Cell lysates of HBEC stimulated with IL-17A/F (50 ng/mL), TNF (20 ng/mL), or IFN-γ (30 ng/mL) were examined for protein abundance by western blots using antibodies specific to the selected candidate APPs. Tenascin could not be validated by western blots due to challenges associated with the antibody reagent. Immunoblots confirmed that the patterns of protein expression profiles of the selected APPs aligned with that observed in the proteomic SOMAscan analyses; lipocalin-2 abundance was enhanced ~10-fold in response to IL-17 alone (Figure 4a,c), and elafin was increased ≥ 5-fold in response to cytokines IL-17 or TNF (Figure 4b,d). In contrast, IFN-γ decreased both lipocalin-2 and elafin protein abundance in HBEC. Cathepsin V protein abundance was decreased in response to all the cytokines i.e, IL-17, TNF and IFN-γ (Figure 5a). Decrease of cathepsin V was modest in response to IL-17, whereas TNF and IFN-γ significantly decreased the abundance of cathepsin V by more that 10-fold compared to unstimulated cells (Figure 5a). In contrast, cathepsin S abundance was significantly enhanced in response to IFN-γ alone compared to unstimulated cells (Figure 5b).

## 4. Discussion

In this study we demonstrate that inflammatory cytokines i.e., IL-17, TNF and IFN-γ result in disparate alteration of APP production in bronchial epithelial cells. These cytokines play an important role in promoting inflammatory processes in the lungs [18,19,20,21,22,23,24,25,26]. IL-17, TNF and IFN-γ are produced by different cell types including immune cells and epithelial cells, and are enhanced in pulmonary inflammatory diseases such as asthma and COPD [34]. One of the mechanisms employed by these cytokines to enhance airway inflammation is by activating the airway epithelium which results in the production of chemokines to increase leukocyte recruitment to the lungs [35,36,37]. Consistent with this, we demonstrate that IL-17 and TNF activate HBEC to induce the production of neutrophilic chemokines, whereas IFN-γ mediates the production of monocytic chemokine. Some studies have suggested that airway inflammation may be a risk factor for increased infections [11,38], and that this may be due to the altered expression of specific APPs [11]. Abundance of several APPs is altered in chronic airway disease such as cystic fibrosis, COPD and asthma. Moreover, specific APPs are elevated in chronic inflammatory diseases such as asthma and COPD and contribute to the exacerbation of these conditions [39,40]. Indeed, mediators of airway inflammation such as air pollution, allergens and smoking have been associated with altered production of specific APPs in the lungs and in bronchial epithelial cells [16,41]. Despite reports demonstrating a strong association between inflammatory mediators and APP expression, the effect of inflammatory cytokines on APP production in the lung remains unclear.

In this study, we show that inflammatory cytokines IL-17, TNF and IFN-γ mediate distinct APP proteomic signatures in HBECs. We demonstrate that cytokines IL-17 and TNF induce similar APPs expression profile. For example, the proteomics screen show that APPs such as elafin, bacterial permeability increasing protein (BPI), angiogenin and antileukoproteinase/SLPI are all altered similarly in response to IL17 and TNF. These findings suggest an overlap between signaling pathways mediated by IL-17 and TNF which result in changes in APPs production. IL-17 is known to functionally–cooperate with TNF and amplify the responses induced by TNF primarily via the transcription factor CCAAT/enhancer-binding protein (C/EBP) [42]. Interestingly, C/EBP has been suggested to be involved in the regulation of expression of specific antimicrobial peptides [43,44]. Thus regulation of expression of APPs that are similarly altered by cytokines IL-17 and TNF such as elafin, as identified in this study, may be controlled by common transcription factors such as C/EBP. The proteomics screen also shows that the expression of certain APPs such as tenascin is enhanced in response to TNF alone, and not IL-17 or IFN-γ. This is consistent with previous studies that have demonstrated enhancement of tenascin levels by TNF in bronchial epithelial cells [45]. Tenascin is known to be enhanced in asthma and is indicative of airway remodeling and fibrosis [45,46]. We also demonstrate that IFN-γ stimulation results in an APP profile that is distinct from that mediated by either IL-17 or TNF. A previous study has shown that IL-17 and IFN-γ differentially regulate downstream responses in synovial fibroblasts [47]. Therefore, we can speculate that IFN-γ-mediated regulation of the APP expression profile in HBECs could be different from that induced by IL-17 or TNF. Overall, the results in this study provide the impetus to further investigate regulatory mechanisms that are similar and distinct in controlling the expression of APPs that are altered by specific inflammatory cytokines.

Independent validation of candidate APPs selected from a proteomics screen show that IL-17 can significantly enhance the abundance of lipocalin-2 and elafin in HBECs. Elafin is also enhanced in response to TNF. However, IFN-γ does not enhance the abundance of lipocalin-2, and significantly decreases elafin abundance in HBECs. Lipocalin-2 is a siderophore-binding antimicrobial protein, and a neutrophil chemotactic factor that is upregulated in epithelial tissues during inflammation [48,49]. This is aligned with the function of IL-17 in promoting neutrophil recruitment, which is mediated through the induction of CXCL1 and CXCL8, in the lungs [50]. Thus, our results suggest that lipocalin-2 may be contributing to IL-17-mediated airway inflammation. Concomitant with this, we have recently shown that inhaled allergen and diesel exhaust, which are known to contribute to airway inflammation in the lungs, enhance the abundance of lipocalins in human bronchoalveolar lavage fluid [15]. Lipocalins exhibit diverse functional roles in the lung, which include contributing to innate immunity for the control of infections, and contributing to the enhancement of airway inflammation, in particular neutrophilic inflammation leading to epithelial damage [49]. In contrast, elafin is an antimicrobial protein that exhibits anti-inflammatory properties [51]. Elafin is known to inhibit serine proteases, such as human neutrophil elastase, thus preventing excessive damage and protecting the airway epithelium during inflammation [52]. Therefore, even though both lipocalin-2 and elafin are antimicrobial proteins, the opposing roles of these proteins in the context of inflammation highlight the complexity of delineating immunomodulatory functions of APPs in airway inflammation.

We demonstrate that cathepsin S abundance is increased, whereas that of cathepsin V is significantly decreased, by IFN-γ in HBECs. Cathepsins are cysteine proteases which are known effectors of tissue remodeling. It is known that IFN-γ regulates cathepsin S expression in the airway epithelial cells and the lung parenchyma in an Interferon Regulatory Factor (IRF)-1 dependent manner [53]. Downstream effects of cathepsin S activity result in apoptosis of epithelial cells [54] and digestion of elastic tissue [55], suggesting that cathepsin S may play a role in tissue remodeling in chronic airway inflammation. Also, cathepsin S is known to degrade specific APPs that contribute to innate immunity such as human β-defensins [56] and SP-A [57]. In contrast, we show that cathepsin V is significantly decreased by IFN-γ. Similar to cathepsin S, cathepsin V also plays a role in airway remodeling [58]. Overall, our study demonstrates that expression of these cathepsins are altered by IFN-γ in HBECs, however the functional relevance of the crosstalk between IFN-γ-mediated immune regulation and specific cathepsin expression remains to be fully delineated.

## 5. Conclusions

The results of this study demonstrate that inflammatory cytokines known to enhance airway inflammation alter APPs expression profiles disparately in bronchial epithelial cells. This suggests that the composition of cytokines within the inflammatory milieu of the lungs may influence relative abundance of specific APPs, and consequently impact the ability to resolve infections. As certain APPs exhibit opposing roles in modulating inflammatory processes, regulation of APPs expression by inflammatory cytokines as indicated by the findings of this study, highlight the complexity of delineating the immunomodulatory functions of APPs in the context of airway inflammation.
